# What was happening in the collaboration between general practitioners and public health nurses in the community: A qualitative study

**DOI:** 10.1002/jgf2.637

**Published:** 2023-06-29

**Authors:** Fumiko Watanabe, Keiichiro Kita, Naoko Kobayashi, Maiko Kuroiwa, Yosuke Shimizu, Azusa Sekijima, Seiji Yamashiro, Daisuke Son

**Affiliations:** ^1^ Toyama City Machinaka Clinic Toyama Japan; ^2^ Department of General Medicine Toyama University Hospital Toyama Japan; ^3^ Nanto Family and Community Medical Center Nanto Japan; ^4^ Kamiichi Sogo Hospital Kamiichi Japan; ^5^ Department of Community‐based Family Medicine, Faculty of Medicine Tottori University Yonago Japan

**Keywords:** general practitioner, interprofessional collaboration, public health nurse, qualitative study

## Abstract

**Background:**

Interprofessional collaboration in the community is becoming essential in primary care, particularly collaboration between public health nurses and general practitioners. However, the precise value of such collaboration has not been sufficiently studied. The purpose of this study was to conduct a qualitative analysis of collaboration between general practitioners and public health nurses in the community to explore the details of the phenomenon and its possible impact on the community.

**Methods:**

Since 2015, The University of Toyama has been implementing the Collaborative Health Activities Project, in which general practitioners and public health nurses work together to promote community health. Focus group and individual interviews were conducted with participating staff, and the data were analyzed qualitatively.

**Results:**

Fifteen themes were generated, in six categories. The categories were as follows: enhanced roles of public health nurses and physicians in the community, new perspectives on the community, public health nurses' sense of trust and empathy toward physicians, bonds of solidarity between public health nurses and physicians, proactive change in residents, and supporting “hangout places”.

**Conclusion:**

The collaboration between general practitioners and public health nurses familiar with the same community fostered a sense of trust and empathy and created the bonds of solidarity between staff and residents. The results also suggest the collaboration may have a positive impact on the local community by inspiring residents to change proactively and supporting “hangouts” where residents and professionals can informally connect.

## Graphical abstract

This study is a qualitative analysis of what was happening in collaboration between general practitioners and public health nurses in a Japanese community. Results revealed the formation of bonds of solidarity between physicians and public health nurses, suggesting that they may have a positive impact on the community.

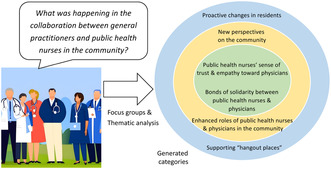

## 1 INTRODUCTION

Interprofessional collaboration is important in primary care these days, given the variety of issues facing communities. It is important to improve patient care quality, practice patient‐centered care, prevent medical errors, and improve patient care outcomes.^1^, ^2^ Underlying the promotion of interprofessional collaboration in health care is the complexity and multifaceted nature of community health needs and the healthcare system. The accumulation of research findings suggests that improved multi‐professional collaboration is essential for providing effective and comprehensive care.^3^

Several studies have already documented the effectiveness of multidisciplinary collaborative practice: an increase of the cost‐effectiveness of health care, the improvement of patients' outcomes and the promotion of satisfaction of both patients and healthcare professionals.^3^, ^4^, ^5^

The quality of physician–nurse collaboration in primary care has many impacts on patients and the community. A study in Belgian primary care evaluated interprofessional collaboration between general practitioners and nurses. It identified a funding system that hinders collaboration, weak functional integration, and lack of interprofessional education as factors that hinder interprofessional collaboration.^6^

There are few studies on collaboration between public health nurses and general practitioners. Collaboration between public health nurses and general practitioners is less common than in other professions. A 2005 study conducted on family physicians in Canada revealed that they collaborated with home health nurses at a rate of 32.0%, whereas their collaboration rate with public health nurses was only 15.0%.^7^ A classic study of collaboration between general practitioners and district health nurses in the United Kingdom found that the attachment of nurses to general practice, the number of general practitioners with whom the health worker worked, and working in the same building were associated with collaboration.^8^ A Dutch study of collaboration between general practitioners and district health nurses in home palliative care suggested that the collaboration was improved by meetings every 6–8 weeks and regular participation by the general practitioners, district health nurses, and a palliative care consultant.^9^ However, the process and effects on the community of collaboration between general practitioners and public health nurses in primary care are not fully understood.

In Japan, public health nurses, unlike other medical professions, are often employed by local governments and engage in community activities involving residents, businesses, and schools. Japanese public health nurses provide a wide range of programs, including family health guidance, support groups, health check‐ups for children and adults, rehabilitation and support for the frail elderly, identification of community health problems, and various health promotion activities.^10^ Municipalities and prefectures employ most public health nurses, and their services are free. In addition, public health nurses interact with all types and ages of people, including pregnant women, infants, children, adolescents, adults, and the elderly. They work with many stakeholders in health prevention to solve health problems and promote health. Their role requires coordination, collaboration, and facilitation.^10^ Collaboration with various professions is an important part of a public health nurse's work to promote primary health care in the community; however, no studies have explored how collaboration with general practitioners affects the community.

The purpose of this study is to explore, through qualitative analysis, the details of what is happening and what is valued during the project in which general practitioners and public health nurses collaborated in a rural area of Japan and the possible impact it had on the community.

## 2 METHODS

### 2.1 A collaborative project between general practitioners and public health nurses at the University of Toyama

Department of Toyama Primary Care, endowed by the City of Toyama and established at the University of Toyama, has been promoting the “Collaborative Health Activities Project (CHAP)” since 2015 to advance collaboration between public health nurses and general practitioners. Three physicians (two certified family physicians and one certified primary care physician) have participated in various public health nursing activities. For example, (1) holding health classes, (2) home visits (postpartum depression mothers, needy people, etc.), (3) maternal and child healthcare events, and (4) public health nurse team meetings and case study meetings (Table 1). A total of 35 activities were conducted by the CHAP during the year from April 2015 to March 2016, and the activities covered 10 districts in the central city under the jurisdiction of the Toyama City Central Health and Welfare Center.

**TABLE 1:** CHAP activities between March 2015 and February 2016.

Contents	Number of times
Health classes	13
Home visits	5
Maternal and child healthcare events	4
Case study meetings	2
Other activities	11

### 2.2 Participants and data collection

This study was based on the constructivist paradigm, which states that human knowledge is not discovered but socially constructed.^11^ Sampling was by purposive sampling. Public health nurses and physicians who participated in the CHAP were invited to join in the focus groups. The questions asked in the interviews were as follows: “What were the positive aspects of the project activities and what effects did you feel?”, “What were the difficulties in the activities and what should we pay attention to in the future?”, and “What impact do you think these activities had on multidisciplinary cooperation and residents?” With permission, we recorded the interviews and prepared a verbatim transcript.

### 2.3 Data analysis

Thematic analysis was used for the data analysis.^12^ Qualitative analysis was chosen because the researchers were also primary care general practitioners and participants in CHAP. They already had a relationship with the interviewees, and the interviews would allow the researchers to fully explore the subjective experiences of the study participants.

The thematic analysis approach follows a six‐step process: familiarization, coding, generating themes, reviewing themes, defining and naming themes, and writing up the results.^12^, ^13^

The first and second authors (FW and KK) interviewed the participants, and the qualitative analysis was primarily conducted by three main authors (FW, KK, and DS). The appropriateness of the themes and the structure between the themes was then reviewed and determined after discussion with the other authors (NK, MK, YS, AS, and SY). The first author (FW) had participated in the CHAP and had a relationship with their interviewees, which may have contributed to their psychological safety during the interview. KK and SY were supervising the project, and although they did not actually participate in the activities. The authors, NK, MK, YS, and AS, were not directly involved in the project, but were general practitioners working in the same area and had discussions during the course of the project.

Following the methodology of qualitative analysis, we conducted iterative data analysis and discussed the validity of interpretations among researchers for triangulation.

### 2.4 Ethical considerations

This study was conducted with the approval of the University of Toyama Ethical Review Committee (Approval No. Hito 28–28). Study participants were informed in advance that their participation in the study was voluntary, and all participants provided their written consent.

## 3 RESULTS

Two public health nurses and two physicians participated in the focus groups. The background of the participants is shown in Table 2. The thematic analysis generated 15 themes and 6 categories (Table 3). The categories were as follows: enhanced roles of public health nurses and physicians in the community, new perspectives on the community, public health nurses' sense of trust and empathy for physicians, bonds of solidarity between public health nurses and physicians, proactive change in residents, and supporting “hangout places.”

**TABLE 2:** Background of study participants.

ID	Profession	Age	Gender	Years of experience	Workplace	Qualification
1	Physician	30's	Male	11	Department of General Medicine, University hospital	Certified family physician
2	Physician	30's	Male	12	Department of General Medicine, University hospital	Certified primary care physician
3	Public health nurse	40's	Female	20	Municipal health and welfare center	Public health nurse, certified social worker
4	Public health nurse	40's	Female	24	Municipal health and welfare center	Public health nurse, registered nurse

**TABLE 3:** Results of the thematic analysis.

Categories	Themes
Enhanced roles of public health nurses and physicians in the community	Outsiders who know the ins and outs of the community
	Active sidestepping
	Catalyst
New perspectives on the community	Enjoyment of working with physicians who bring new perspectives
	Sense of accomplishment through health activities that capture the true feelings of residents
Public health nurses' sense of trust and empathy toward physicians	Sense of trust in consulting without barriers
	Sympathy among people who know the same community
Bonds of solidarity between public health nurses and physicians	Mutual interaction as a source of vitality
	Cooperation among those who coordinate the entire region
	Harmonizing of two parties engaged in an activity
Proactive changes in residents	Residents' proactive participation in activities
	Change in residents' awareness from self‐help to mutual help and assistance
	Increased satisfaction and enjoyment of residents
Supporting “hangout places”	Fostering a “hangout” in the community through health activities
	Existence of a “place” that occurs naturally

We describe each category below, citing representative texts.

### 3.1 Enhanced roles of public health nurses and physicians in the community

Regarding the roles of public health nurses and physicians in the community, they saw themselves not as the main actors but as supporting actors. In this role, they aimed to function as catalysts bringing about changes in residents' awareness and behavior. They also felt that as “outsiders” who knew the inner workings of the community well, they were free from community ties and restrictions and could easily fulfill the function of a catalyst.

We should be the followers, not the masters. We can connect and make them independent. … (We are) gofers. Yes. Well, the residents and patients are the main actors, as long as we keep the stance of helping them to do well, even if the person in charge changes. (Theme: active sidestepping)

### 3.2 New perspectives on the community

The public health nurses found the new perspectives of the general practitioners on the community interesting and enjoyed collaborating with them. They also felt a sense of accomplishment through repeated trial‐and‐error practice, using the new perspectives to determine how best to develop health activities that captured the needs of the residents and recognized their true feelings.

When I first experienced the work of listening to local residents together, I had a different perspective from the way Dr. K felt and organized the conversation, and I think I found it interesting. That's why I thought it would be good to work together with him from the planning stage. (Theme: enjoyment of working with physicians who bring new perspectives)

I had originally wanted to do something that involved the participation of residents, and I thought this was a way to develop it. It was a process of trial and error. We have been trying out new styles, or rather accumulating new styles, and have come to feel that this form seems to be a good one. (Theme: sense of accomplishment through health activities that capture the true feelings of residents)

### 3.3 Public health nurses' sense of trust and empathy toward physicians

The public health nurses empathized with general practitioners who have a comprehensive view of the community and its residents and felt comfortable consulting with them without any barriers. The public health nurses felt a sense of sympathy among people who know the same community because they share the same values and see the same region.

I guess it's not just a collaborative activity between doctors and public health nurses… It may be too strong to call it a belief, but if you don't share the values of the person and the district, it won't work, and in fact, even at the same health center, it won't work with people who don't share those values. (Theme: sympathy among people who know the same community)

### 3.4 Bonds of solidarity between public health nurses and physicians

Public health nurses and physicians, with shared values as generalists with a comprehensive view of the community and people were empowered by their interactions as a source of vitality. They also felt a sense of solidarity with those coordinating the entire community, which facilitated collaboration. This was the “A‐Un” spirit (harmonizing of two parties engaged in an activity), where people could communicate with each other even without exchanging words.

Of course, the activities were aimed at the residents, but the collaboration itself became so enjoyable that we were doing it for our own good. (Theme: mutual interaction as a source of vitality)

When there is a great deal of collaboration, even if we don't meet, we can somehow see how things are going to turn out. Then, if you talk to them a little, you can just say, ‘That's the way it's always done,’ and it's over.(Theme: harmonizing of two parties engaged in an activity)

### 3.5 Proactive changes in residents

The public health nurses and physicians felt that the collaborative project had made the residents more proactive. They felt that the focus of motivated residents changed from self‐help to mutual assistance, which also increased their sense of satisfaction and enjoyment.

I think they are aware of it. I think they are proud to say, ‘We are doing it ourselves!’ Even if we leave them alone, they are moving on. That's a very big part of it. I was glad that I put my foot and hand into it. (Theme: residents' proactive participation in activities)

### 3.6 Supporting “hangout places”

Physicians and public health nurses felt that the mutual interaction in collaborative activities itself is a source of vitality and that these health activities themselves have the same function as that of a “hangout” where anyone can casually attend and be present in the community. They hoped that the increase in resident‐led activities would lead to a spontaneous increase in the number of such “hangout” places.

Even if they had no special purpose of being there, their interaction with the public health nurses may have energized them. Just as a ‘hangout’ did. (Theme: fostering a “hangout” in the community through health activities)

I think it would be better if they became proactive in that way, even when we don't encourage them to join us every time. I hope they will get together spontaneously. The circle A is active by setting a date for the next meeting each time. Some gatherings are spontaneous. (Theme: existence of a “place” that occurs naturally)

## 4 DISCUSSION

In this study, we analyzed what was happening in the collaboration project between general practitioners and public health nurses and what the possible impact it had on the community. As a result, there was an awareness between physicians and public health nurses of the supporting and catalytic roles of public health nurses and physicians, the new perspectives in looking at the community, the sense of trust and empathy of public health nurses toward physicians, and the bonds of solidarity and harmonizing spirit between public health nurses and physicians. In addition, as an impact on the community, it was suggested that residents may have proactively changed and that health activities may have functioned as a local hangout.

A Lithuanian study on the collaboration between general practitioners and community nurses indicated that individual commitment as part of a team based on trust and respect among the members was important to create synergy.^14^ This is similar to the results of this present study: public health nurses felt secure and empathetic toward physicians, which created the bonds of solidarity. The Lithuanian study also suggested the importance of the relational communication, that is, not what to say but how to say, between public health nurses and physicians.^14^ This type of communication is useful not just to share information, but to build good relationships with each other and to improve the atmosphere of the team. This study also found that a secure communication style based on mutual relationships, such as harmonizing spirit, is important.

Although not a study on public health nurses, an Australian study has shown that collaboration between clinic‐based nurses and general practitioners effectively identified a variety of unmet physical and psychosocial needs in the elderly that could be addressed to improve their quality of life.^15^ The present study also showed a sense of accomplishment in public health nurses who incorporated new perspectives from general practitioners while capturing the true feelings of the residents.

The role of “outsiders” in community development has been discussed in the past. Rural development projects in developing countries often come from outside experts and researchers. However, they do not foster a drive for sustainable development in the local people, limiting the sustainability of their achievements.^16^ Community members should make their own decisions, while receiving support from outsiders in order to tackle their own problems.^16^, ^17^ Similarly, the present study discussed the importance of outsiders, such as public health nurses and physicians, playing a supporting and catalytic role, rather than being the main actors.

What can be pointed out to be a novelty of this study is that we have found an importance of the local hangout, where residents and professionals can loosely connect. While past social capital studies have discussed the difference between loose and strong ties,^18^, ^19^ a study in Australia found that residents trusted professionals but wanted loose ties with them.^20^ Stronger ties can provide a variety of social support but loose ties function as a bridge between social sources creating the potential for diverse resource solutions to complex challenges. Research in Japan also suggests that creating loose community connections can create diverse encounters among residents and transform them into proactive actors.^21^

The small sample size of this study is one of the limitations. We may have missed some perspectives on the collaborative activities of public health nurses and general practitioners. In addition, the participating public health nurses may have had favorable opinions on the activity and the physicians, which could discourage the discussion of any negative aspects of this activity. It should also be noted that the results regarding the impact on the residents were perceived subjectively by the public health nurses and physicians and were not the results narrated by the residents themselves.

## 5 CONCLUSION

The study revealed that general practitioner and public health nurse collaboration in the community has brought about the following: enhanced the roles of public health nurses and physicians in the community, widened the perspectives on the community, engendered a sense of trust and empathy in public health nurses toward physicians, and fostered the bonds of solidarity between them. As for the possible impact on the community, it was suggested that the project could inspire residents to change proactively and set up “hangouts.”

## AUTHOR CONTRIBUTIONS

FW and KK interviewed participants and were major contributors to analyzing the text data under the supervision of DS. The results of the analysis were discussed with YS, AS, MK, NK, and SY for triangulation and the results were brushed up. FW mainly wrote the manuscript with the assistant of DS. All authors read and approved the final manuscript.

## FUNDING INFORMATION

The authors have not received any funding for the current study.

## CONFLICT OF INTEREST STATEMENT

The authors have stated explicitly that there are no conflicts of interest in connection with this article.

## ETHICS STATEMENT

This study was conducted under the approval of the University of Toyama Ethical Review Committee (Approval No. Hito 28‐28).
